# Neuronize: a tool for building realistic neuronal cell morphologies

**DOI:** 10.3389/fnana.2013.00015

**Published:** 2013-06-03

**Authors:** Juan P. Brito, Susana Mata, Sofia Bayona, Luis Pastor, Javier DeFelipe, Ruth Benavides-Piccione

**Affiliations:** ^1^Universidad Rey Juan CarlosMadrid, Spain; ^2^Laboratorio Cajal de Circuitos Corticales, Centro de Tecnología Biomédica, Universidad Politécnica de MadridMadrid, Spain; ^3^Instituto Cajal (CSIC)Madrid, Spain; ^4^Centro de Investigación Biomédica en Red sobre Enfermedades NeurodegenerativasMadrid, Spain

**Keywords:** pyramidal cells, virtual dendrites, morphology simulation, dendritic structure, 3D models, multiresolution visualization approach

## Abstract

This study presents a tool, Neuronize, for building realistic three-dimensional models of neuronal cells from the morphological information extracted through computer-aided tracing applications. Neuronize consists of a set of methods designed to build 3D neural meshes that approximate the cell membrane at different resolution levels, allowing a balance to be reached between the complexity and the quality of the final model. The main contribution of the present study is the proposal of a novel approach to build a realistic and accurate 3D shape of the soma from the incomplete information stored in the digitally traced neuron, which usually consists of a 2D cell body contour. This technique is based on the deformation of an initial shape driven by the position and thickness of the first order dendrites. The addition of a set of spines along the dendrites completes the model, building a final 3D neuronal cell suitable for its visualization in a wide range of 3D environments.

## Introduction

Over the last few decades, the development of new computational methods and tools has significantly contributed to advances in the study of brain structure and function. Outstanding contributions have been made to the field of complex systems using visualization-based approaches, which have been successfully applied to the design and analysis of a variety of complex systems, exploiting the ability of the human visual system to extract information from visual scenarios (Kikinis et al., 1996; Harb Manssour and Sasso Freitas, 1999; Lempert, 2002; Barnes and Shaw, 2009; Rubinov and Sporns, 2010). In particular, visual analytics or data visualization are extremely active, multidisciplinary research fields focused on providing graphical representations of data that increase the understanding of the phenomenon being observed (Preim, 2007; Linsen et al., 2008; Barnes and Shaw, 2009). Specifically, the benefits of incorporating visualization-based approaches into the analysis of complex systems have already become evident in neuroscience research. For example, a method based on the implementation of straightening and unrolling transformations has been recently developed to transform the previously-analyzed 3D structure of spine insertions in dendrites of pyramidal cells to a planar, unfolded arrangement (Morales et al., 2012). The implementation of these visual technologies facilitates the analysis of neuron structure, providing new insights into the organization and distribution patterns of these cells.

At present, there is a wide variety of software tools that help in various tasks such as segmentation, visualization, simulation, etc. (reviewed in Meijering, 2010). In particular, the digital reconstruction of neural cells from a stack of images can be achieved with an automatic or semi-automatic process consisting of an initial segmentation step followed by the extraction of an isosurface that approximates the cell membrane. This process provides an accurate representation of the real shape that can be reconstructed from a confocal microscopy stack of images using specific tools for thresholding and isosurface extraction (e.g., Imaris Software, 2012). However, segmentation of complex images requires sophisticated and domain-specific solutions that are not easily automated. For this reason, neural reconstructions are usually obtained by means of computer-aided tracing applications (e.g., Neurolucida). Briefly, the morphological data extracted with these applications typically consist of a set of points that trace the skeleton of the neuron, capturing the shape and the trajectories of the dendrites/axon in 3D, which allows for the study of several morphological parameters that include features of the dendrites/axon and soma. However, regarding the cell body shape, the extracted information consists only of a set of connected points tracing the contour of the soma in 2D. Previous studies sometimes ignored the soma and, at best, represented it with a simple sphere, which does not represent the real morphology of the cell. To our knowledge there are no software packages available that provide a 3D reconstruction of the soma shape from computer-aided tracings, where the information describing the soma is not sufficient to recover its 3D shape. Therefore, the goal of the present study was to develop a new technique specifically focused on building 3D polygonal meshes which approximate the shape of somata, using as a starting point the incomplete morphological data extracted by computer-aided tracing applications. Our particular technique uses mass-spring system (Terzopoulos et al., 1987; Erleben et al., 2005), since it is considered one of the simplest deformable models (Nealen et al., 2006) developed by the Computer Graphics community. We also build high quality meshes of dendritic and axonal arbors, including detailed reconstruction of dendritic spines (for simplicity, spines). These algorithms have been integrated into a tool, called Neuronize that is publicly available at http://cajalbbp.cesvima.upm.es/neuronize. The different meshes are built and connected to form a smooth mesh suitable for visualization, and for applying standard and advanced graphical algorithms of texture mapping, shading or geometry processing (Luebke, 2001; Gu et al., 2002; Sorkine et al., 2002). Finally the possibility of extracting the mesh at different levels of resolution provides models suitable for multiresolution visual approaches (Clark, 1976; Luebke et al., 2002).

## Methods and results

**Figure 1:**
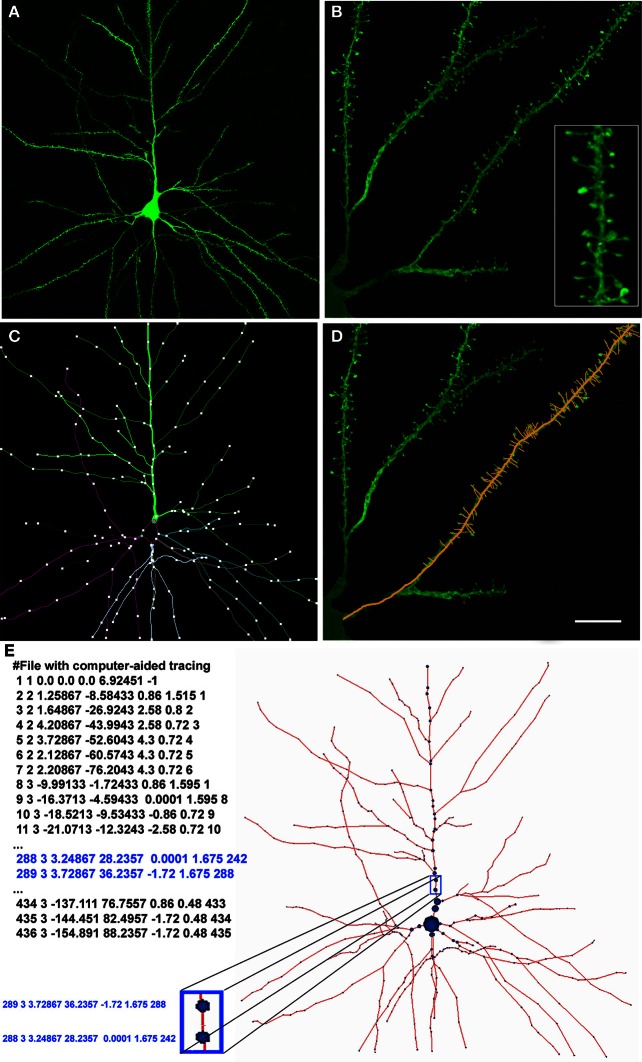
**(A)** Confocal microscopy image of an intracellularly injected layer III pyramidal neuron of the human cingulate cortex. **(B)** High magnification image showing basal dendrites and spines (inset). **(C)** Computer-aided tracing in 3D (Neurolucida) of the neuron shown in **(A)**. **(D)** Same image illustrated in **(B)** showing a superimposed 3D tracing of the positions of the dendritic spines. **(E)** Digital representation of the pyramidal neuron shown in **(A)** and **(C)**. *Left:* the neuron definition where each row represents a morphological point (identifier, type of point, (X,Y,Z) coordinates, radius at that point, and parent identifier). *Right:* depiction of the morphological skeleton defined on the left. Scale bar in **(D)**: 50 μm in **(A)**, 10 μm in (**B, D**) (4.5 μm inset).

This study presents a set of methods designed to build 3D neural models, taking the morphological information extracted through computer-aided tracing applications as the starting point. The input data obtained from biological experiments consist of a set of points that define the shape of pyramidal neurons, the most frequent cortical neuronal type. These neurons were intracellularly injected with Lucifer Yellow (LY) in layer III of the human cingulate cortex from a 40-year-old human male obtained at autopsy (2–3 h post-mortem) and subsequently stained for LY (see Figure 1A; further information regarding tissue preparation, injection methodology, and immunohistochemistry processing are detailed in Benavides-Piccione et al., 2012). Sections were imaged with a Leica TCS 4D confocal scanning laser attached to a Leitz DMIRB fluorescence microscope. Fluorescent labeling profiles were imaged, using an excitation wavelength of 491 nm to visualize Alexa fluor 488. Stacks of images (×20), which included apical and basal dendritic arbors, were acquired. Additional stacks of images, at high magnification (×63 glycerol) were also scanned to capture spines (Figure 1B).

Data points of neuron morphology were extracted using Neurolucida Confocal package (MicroBrightField; Figures 1C–E). Briefly, the soma was defined through a set of connected points tracing the contour of the soma in 2D. Dendrites/axon in the skeleton definition were described through 3D points, delimiting the different segments that form the dendritic arbor. These points have an associated diameter that provides the information of the varying thickness of the dendrite at that particular point, and varies along the length of the dendrite. Spines were separately reconstructed at higher magnification along the length of the dendrite obtaining 3D points that delimited the position of spines (Figure 1D).

To implement the techniques included in Neuronize, we selected the C++ programming language and Matlab 7.12. (2011). For the building of the model, the auxiliary libraries OpenMesh (Botsch et al., 2002), Boost (Boost C++ libraries 1.50, 2012), Qt SDK (Qt SDK 4.8, 2012), libQGLViewer (libQGLViewer, 2012) and Peyré toolbox for Geodesic Computations on 3D Meshes (Peyré and Cohen, 2005) were used. The 3D building of neurons is structured according to the following stages:

### Building the soma

The incomplete information stored in the digitally traced neuron usually consists of a 2D contour that approximates the shape of a 2D projection of the cell body. Some laboratories store only a point defining the soma center and a radius approximating its size. Thus, there is not enough information to recover the original 3D shape in either of these two cases. Our proposal calculates the deformation that an initial sphere would undergo when simulating the dendrites pulling away from the sphere, until the cell body reaches its final growth state. For this purpose, we used a technique based on a mass-spring deformation algorithm (Terzopoulos et al., 1987; Nealen et al., 2006). This technique was applied to create a structure of springs based on the edges of the triangles of the initial 3D sphere, and a set of internal springs from the center of the mesh to prevent the soma from collapsing and to guarantee the specified minimum volume. In this approach, the cell body was modeled as a set of nodes (point masses) connected by springs that obey Hooke's Law (Nealen et al., 2006). We then applied Newton's second law to the point masses including the forces applied by the springs. Hence, we obtained a system of differential equations for the motion of the nodes, which could be solved by standard numerical schemes. Specifically, three different explicit integrators were implemented: Euler, Runge–Kutta 2, and Verlet (Verlet, 1967; Volino and Magnenat-Thalmann, 2001). The Verlet method was selected to generate the results presented in this paper, since it is a fourth order integrator with a computational cost of a second order one. We approached the simulation as an energy minimization problem since the physics of surfaces state that a constrained surface will assume the deformation that minimizes the total energy of the deformation (Terzopoulos et al., 1987). This technique can also be used by laboratories that only store a point defining the soma center and a radius approximating its size since 2D traced contours can be converted to a center and a radius. This simpler morphological definition can then be used as the input data of our algorithm.

**Figure 2:**
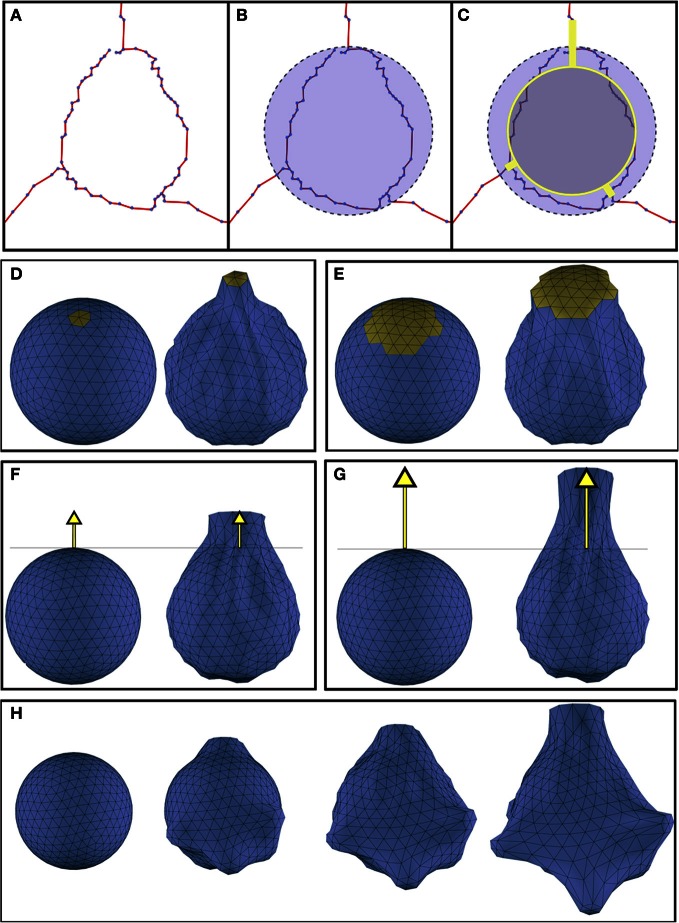
**(A)** Initial morphological definition of a soma. **(B)** The bounding sphere of the soma. **(C)** In gray, the reduced sphere, and in yellow, the initial dendritic segments used to carry out the deformation process. **(D,E)** Effect of varying the magnitude of the region of influence (yellow): **(D)** region of influence with geodesic distance = 1.39; **(E)** region of influence with geodesic distance = 2.39. **(F,G)** Effect of increasing the displacements of the vertices pulled by the dendrite: **(F)** distance of the vertices representing the starting segment of the dendrite = 1.5; **(G)** distance of the vertices representing the starting segment of the dendrite = 2.5). **(H)** Progressive evolution of the soma deformation process step by step. Deformation parameters were extracted from the morphological data of a real soma.

The building of the soma only requires the definition of the initial segments of the first-order dendrites, and the center and radius that approximate its bounding sphere. The first step of the technique performs a reduction of this initial sphere, thereby establishing the minimum volume of the final deformed soma Figures 2A–C illustrates this process.

This reduced sphere will be represented by a 3D polygonal mesh, which is deformed simulating dendrites pulling towards them. The deformation process behavior mainly depends on two factors:

1. The region of influence of each dendrite: This region is determined by the set of vertices that will be pulled by each dendrite. The region of influence of a dendrite is computed according to its thickness. The closest vertex of the sphere to the starting point of the dendrite is selected and all the neighboring vertices that are within a distance less or equal to the dendrite diameter are included in its region of influence. This distance is computed as a geodesic metric (Peyré and Cohen, 2005). Figures 2D,E shows two different deformations obtained when varying the region of influence of a dendrite.
2. The displacement or final position reached by the vertices under the dendrite's region of influence: The displacement of these vertices induces a force that propagates over the mesh and results in the final deformed shape. The final position of the vertices pulled by a dendrite is selected to be the same as the position of the vertices representing the starting segment of the neurite. Figures 2F,G illustrates the effect of varying this final position.

These two factors, together with the reduction percentage of the initial sphere, allow a wide variety of criteria to be taken into account during the deformation process. Once our mass-spring model is built according to the methodology described, we deform the model pulling toward the beginning of each first order dendrite. Figure 2H depicts the deformation process from an initial sphere at different time steps.

**Figure 3:**
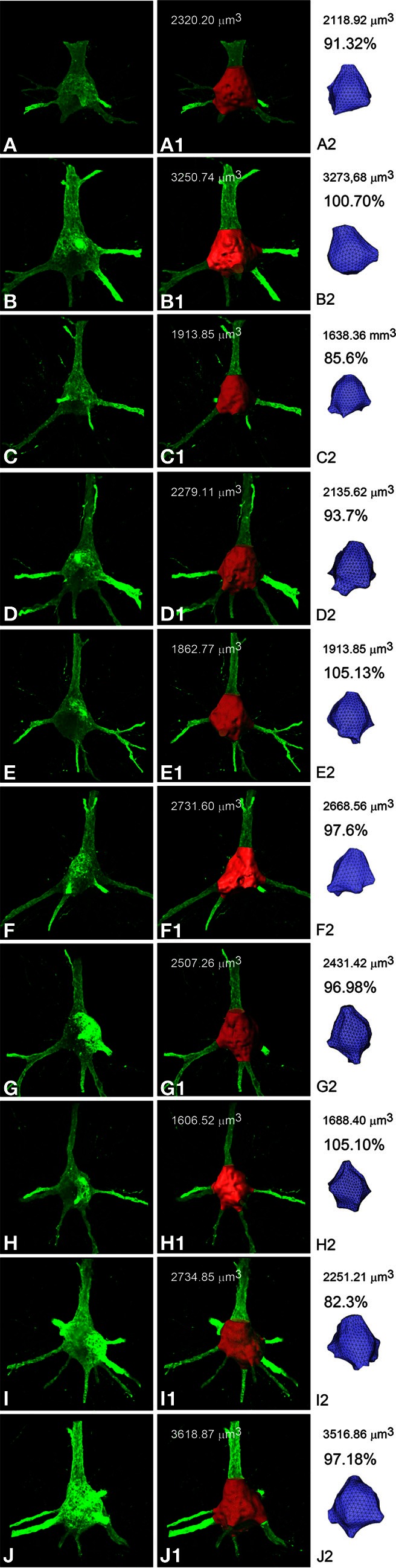
**Confocal microscope stack of images of 10 different somata from layer III pyramidal neurons of the human cingulate cortex (A–J) to compare (percentages) the volume (μm^3^) of the corresponding simple isosurface mesh obtained from thresholding using Imaris Software (A1–J1) with the volume of the corresponding soma meshes obtained using mass-spring system deformation techniques (A2–J2)**.

In order to estimate how similar the real soma and the soma mesh were to each other we quantified the volume of several soma meshes and compared it with their corresponding simple isosurface mesh obtained from thresholding. For this purpose, Imaris Software 2012) (Bitplane AG, Zurich, Switzerland) was used to obtain detailed information from the soma generated by thresholding in order to compare the quality of the built 3D model with the original cell. Figure 3 illustrates how the comparison of the real somas and those obtained using mass-spring system deformation techniques present similar values.

**Figure 4:**
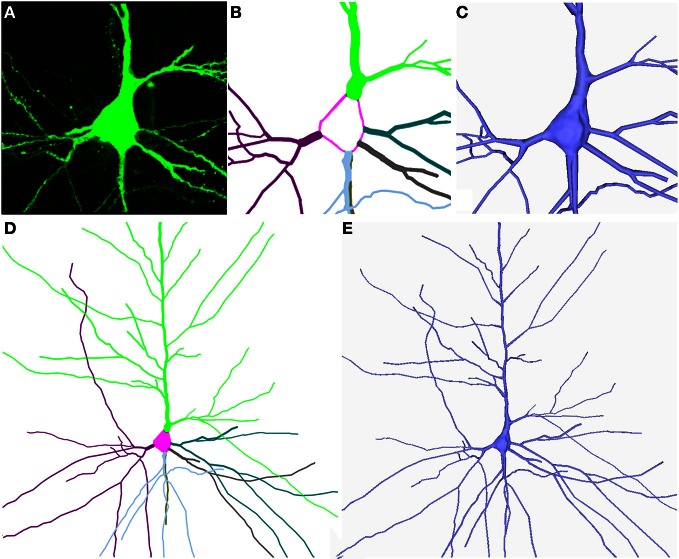
**(A–E)** Shows the building of the soma and the dendrites/axon from the neuron shown in Figure 1. The information (starting points and thickness of the dendrites and the center and radius of the soma) was extracted from the digitally traced neuron shown in Figure 1.

When the simulation finishes and the soma has a plausible shape, a final process is still necessary in order to facilitate the joining of the dendrites and the soma. The aim of this process is to slightly re-arrange the positions of the vertices belonging to the regions of influence in such a way that the diameters of these regions are equal to the diameter of their corresponding first order dendrite. In this process, the algorithm selects the vertices in the contour of the region of influence and displaces them until the region's diameter matches the initial thickness of the corresponding first order dendrite. Later, a fan tessellation between the vertices of the contour and the central vertex is applied, and the rest of the vertices and triangles on the surface (which are no longer needed) are erased. This process makes possible a high quality connection between the deformed soma and each first order dendrite (Figures 4A–C).

### Building dendrites/axons

**Figure 5:**
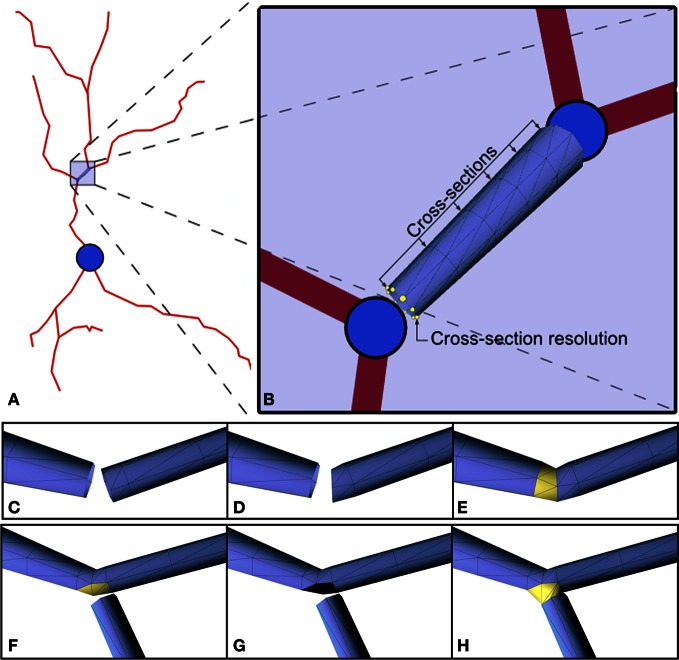
**(A,B)** 3D approximation of the highlighted segment with a truncated cone. The radii of the bases are the radii defined for the morphological points at the extremes of the segment. There are two user-defined parameters: the number of cross-sections between the two bases (6 in this example), and the number of points used to approximate the circular shape of the bases (8 in this example). **(C–H)** Link between elements. Changes in direction of consecutive segments imply that perpendicular sections of the mesh may intersect. The shortening of the first segment solves this problem **(C–E)**. The process of connecting child dendrites at a fork point is shown in the sequence of images illustrated in **(F–H)**.

This step builds 3D triangular meshes that approximate the cell membrane of the dendritic and axonal arbors (Figures 4D,E). Commercially-available software usually build a truncated cone geometry for each segment (see Figures 5A,B), where the segment defines the central axis of the cone and the diameters of the end points define the size of the bases. In this work the resolution of the cone is given by two user-defined parameters: the number of cross-sections between the two bases and the number of points used to approximate the circular shape of the bases. The diameter of any intermediate cross-section is consequently calculated through interpolation from the diameters of the cone's bases according to the position of the cross-section along the central axis segment. Cross-sections are not equally distributed along the segment, but carefully placed to avoid problems when linking or connecting adjacent truncated cone structures. Thus, the definition of resolution-related parameters guides the triangulation of the cones, giving rise to a multiresolution approach that allows the level of detail of the final mesh to be controlled. Finally, connection strategies were designed in order to produce a smooth and continuous mesh of the dendritic and axonal arbors with high quality joints at bifurcations and ensure appropriate connection to the soma.

#### Linking the different meshes

This step of the algorithm links the different built meshes to form a complete neuron mesh. Three different cases can be distinguished: linking consecutive truncated cones, linking at fork points, and linking the first order dendrites with the soma.

For each pair of consecutive truncated cones, we connect them by triangulating the points defining the last cross-section of the first truncated cone with the points defining the first cross-section of the second one. Intersections between consecutive truncated cones are avoided by shortening the first cone and re-orienting the initial base of the second cone (Figures 5C–E). In the case of fork points (bifurcations), we first connect the father segment with the first child segment, following the strategy explained in the previous paragraph. The second child is then connected by opening a hole in the mesh and sewing the first section of this second child segment on to the vertices belonging to the boundary of the hole. This hole is opened around the vertex that is selected as the closest to the centroid of the starting cross-section of the second child branch. Intersections with this second child are avoided by displacing the initial base of the second child branch (Figures 5F–H). In order to join the soma with the tubular geometries of the first order dendrites, we first select the vertex of the soma which is the nearest to the barycenter of the first cross-section of the tubular geometry to be joined. Then, we erase all the triangles connected to that vertex. Note that, as previously mentioned, these triangles form a triangle fan connected to a central vertex. The erasing of these triangles opens a hole in the soma mesh, and the vertices of the contour of the hole in the soma are adapted to be connected to the vertices of the first cross-section of the dendrite. The polygonal mesh obtained at the end of this stage forms a close surface that correctly approximates the cell body and the dendritic and axonal arbors. At this point, a smoothing step can be applied to improve the appearance of the 3D cell membrane.

### Adding spines to the mesh

This phase distributes a set of spines along the dendrites, creating a complete 3D model of the pyramidal cell. In order to distribute these 3D spine models along the mesh, two factors are taken into account: the spine distribution and spine morphology. The tool allows users to load their own dataset of spine distribution. Also, the spine distribution can be computed according to a default dataset of spine densities that is available for modeling, based on previous studies. Indeed, the tool currently includes a default dataset of spine densities for layer III pyramidal neurons of the human cingulate cortex (Benavides-Piccione et al., 2012), and has been designed to include spine distribution of different species/cortical regions and layers in the near future. Similarly, the morphology of the spines can also be modeled if users provide their own dataset, as long as these data have been previously generated through segmentation methods. Again, the tool currently includes a default dataset of spine morphologies (Benavides-Piccione et al., 2012), and has also been designed to include spine morphology of different species/cortical regions and layers.

**Figure 6:**
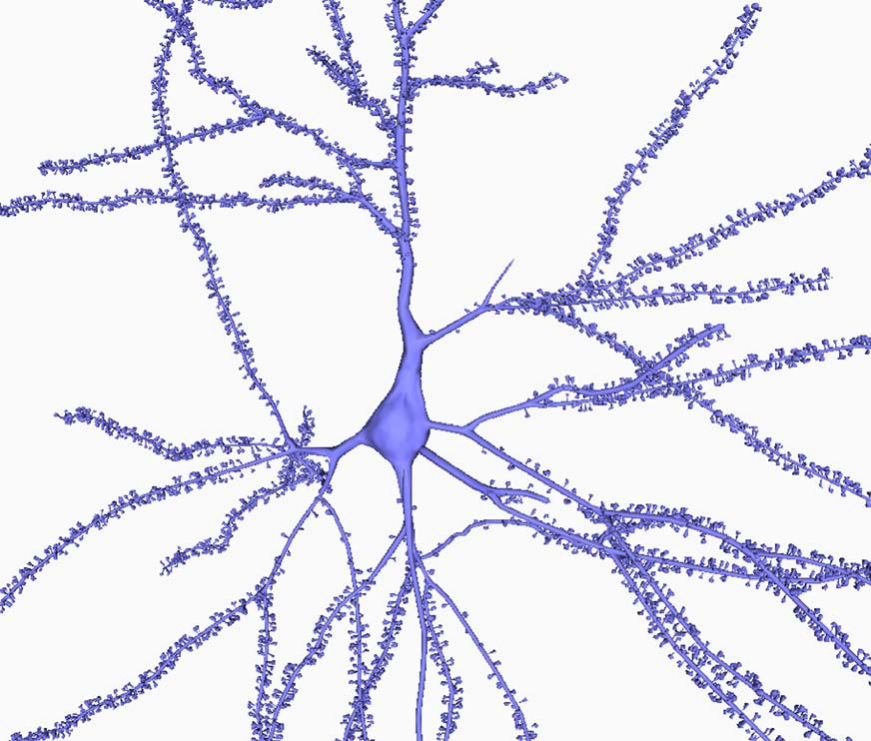
**A general view of a 3D built cell**. Note the realistic shape of the soma and of the dendritic shafts and spines.

In summary, Figure 6 shows the final result of building a 3D mesh using Neuronize. This neuron shows a plausible soma, properly connected dendritic and axonal arbor, and spines distributed along the dendrites. Neuronize performs all the steps of the process: the building of the soma, dendrites/axon and spine density distribution and morphology (if required). The tool includes a graphical interface that presents the standard menu options to open.asc/.swc files and save the built models, making the application intuitive and easy to use. It also gives user the option to parameterize some factors to control features such as the desired final quality of the model or the smoothness of the mesh, while interactively visualizing the obtained result.

## Discussion

The Neuronize tool presented here was conceived to overcome the problems caused by the unrealistic somata and low quality unconnected 3D meshes which are typically generated with other existing neuronal tools based on computer aided tracings. In particular, the method and techniques presented in this tool allow the quick and simple building of accurate tridimensional polygonal meshes of neuronal cells from the incomplete morphological information extracted through computer-aided tracing applications. Thus, the method proposed in this paper is not intended to start from a stack of images, where a simple isosurface mesh that approximates the soma, can be obtained from thresholding. Instead, this technique is designed to work from neuronal tracings where there is not enough information describing the soma to recover its original 3D shape. This technique represents an innovative approach for the construction of somas. We propose a new technique, deforming an initial shape, in a 3D manner, in relation to the dendrites that come out from the soma. This novel deformation strategy builds plausible shapes based only on the starting segments of the dendrites and an estimation of the soma diameter. 3D meshes are built according to the original dendritic and axonal arbors described in the morphological data, presenting high fidelity to the original data. Given that the real somas and the 3D models are rather similar (Figure 3), the deformation approach used in the present study proved itself to be a successful technique to build accurate somata from incomplete morphological data.

**Figure 7:**
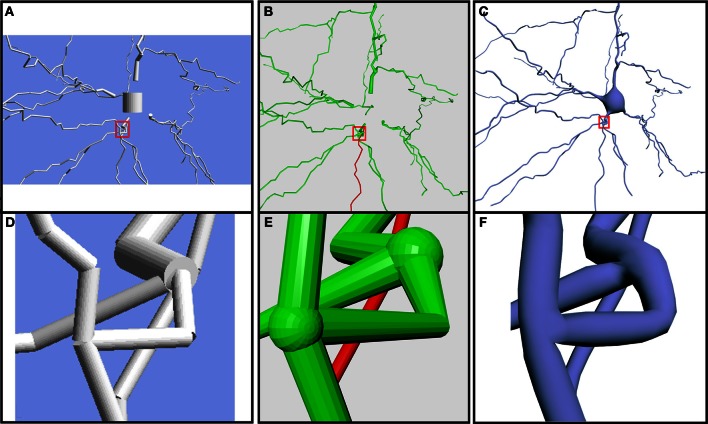
**Comparison of the same neuron using different 3D representations**. Models generated using NeuroConstruct **(A,D)**, Neurolucida **(B,E)** and our proposed method **(C,F)**. Note the unconnected elements and the absence of a realistic soma in **(A,B). (D–F)** Close-up view of the same bifurcation with the three different technologies.

Additionally, the built meshes overcome some limitations of previous neuronal tools that created unconnected, open, or fixed resolution 3D meshes, with unrealistic approximations of the somas' shapes. Existing broadly-used software packages, such as Neurolucida (Glaser, 1990), NeuroConstruct (Gleeson et al., 2007) and Genesis (Wilson et al., 2007), create very low quality meshes and, in some cases, there are parts of the neuron that are unconnected (Figure 7). As it can be observed, NeuroConstruct builds unconnected meshes and the soma shape is approximated with a cylinder. In the case of Neurolucida, the mesh is also unconnected, although this is masked to an extent by an overlapping sphere in each bifurcation. Neurolucida approximates the soma with a 2D disc that is not even saved when the 3D model is exported. Other methods such as the method presented in Lasserre et al. (2011) are able to obtain a smooth representation, but the preservation of desirable properties of the mesh for visualization purposes, such as being 2D-manifold or closed, are not stated as objectives in their work. Additionally, widespread software packages, such as Blender, an application that provides general modeling techniques, can be used to obtain the truncated cones that approximate the dendritic segments. However, this would not directly achieve the linking of cones at the bifurcations, or the distribution of spines and, notably, would not obtain a realistic shape of the soma.

Our tool overcomes these limitations offering multiresolution, closed, 2D-manifold meshes. These characteristics make it easy to process the geometry of the meshes and make them suitable for the application of other visualization techniques (such as subdivision and smoothing algorithms). The meshes accurately follow the morphological descriptions and include realistic somas, specifically built for each cell.

**Figure 8:**
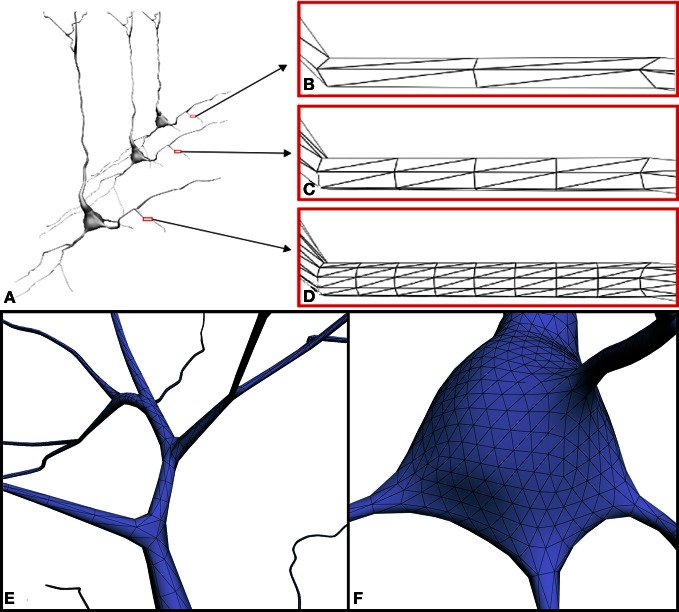
**(A)** Three versions of the same neuron with different resolution levels: **(B)** 2 cross-sections × 3 points per section (4892 triangles), **(C)** 4 cross-sections × 6 points per section (24412 triangles) and **(D)** 8 cross-sections × 12 points per section (98652 triangles). Note that, when viewed from a distance, low-resolution models still provide good visual quality. **(E,F)** Close-up view of the mesh at connecting points: **(E)** example of a branch, **(F)** example of the connection between a dendrite and the soma.

In the present study, the construction process itself guarantees that the dendritic/axonal arbors preserve the original trajectories and diameters extracted from the original tracings. In contrast with other existing tools, the resolution or level of detail of the built mesh can be parameterized, allowing the creation of different versions of the same neuron, each one with a certain degree of detail (Figures 8A–D). This allows accuracy requirements to be taken into account and facilitates future multiresolution visualization approaches. Special attention was devoted to the bifurcations and to the connections of the cell body with the first order dendrites and the axon (Figures 8E,F). However, even at a low level of resolution, the quality of the meshes could be considered sufficient for a general exploration of the neuron and for certain modeling such as voltage-based modeling (e.g., Hay et al., 2011). Higher resolutions may be required for closer inspection or other purposes, such as reaction-diffusion modeling of calcium dynamics (e.g., Wils and De Schutter, 2009).

In addition to the construction of a plausible 3D soma and the building of the dendritic and axonal arbors of a neuron, the techniques that we have developed also give the option to distribute spines along the dendrites. It is possible to do this using a default dataset of spine densities that is provided by the tool. Furthermore, it is also possible to specify the morphology of the spines' population, since the tool also provides a default dataset of spine morphologies. To our knowledge, the possibility of adding spines is not available in any previous neuronal tool. However, it is important to bear in mind that (unless provided by the user) spine density distribution/morphology does not represent the actual position/shape of each spine in the dendrite. Instead, it shows the mean density distribution/shape of spines along the dendrites of the specie/cortical area/layer of the neuron being modeled.

## Conclusions and future directions

To our knowledge, this is the first time that it has been possible to represent real neurons in 3D with plausible somas and with spines along their dendritic arbors (see Figure 6). This opens up new opportunities for studying and analyzing the morphology of the neurons, and provides a tool to easily build a high quality 3D mesh of neurons from its morphological description using standard computer-aided tracing applications, such as Neurolucida.

Future work will study the use of additional criteria to deform the soma and the definition of new error measures in order to further validate the dendritic trees and the soma shape. A rigorous assessment of the accuracy of the results will allow the 3D meshes to be used for other purposes, such as functional modeling and simulations. Additionally, the tool will include a variety of datasets of spine densities and morphologies including different species/cortical regions and layers. A further step toward the interactive visualization of neuronal data will rely on the ability to instantaneously build the 3D models as the user goes along, avoiding in this way the need of explicitly storing the vast amounts of information associated with 3D mesh representations. The algorithms that we have developed are designed to be easily parallelizable; the parallelization of the proposed techniques will result in a meaningful reduction of the computation times.

### Conflict of interest statement

The authors declare that the research was conducted in the absence of any commercial or financial relationships that could be construed as a potential conflict of interest.
